# Reduced HIV symptoms and improved health-related quality of life correlate with better access to care for HIV-1 infected women: the ELLA study

**DOI:** 10.7448/IAS.17.4.19616

**Published:** 2014-11-02

**Authors:** Robert Baran, Fiona Mulcahy, Ivanka Krznaric, Antonella d'Arminio Monforte, Anna Samarina, He Xi, Isabel Cassetti, Jose Valdez Madruga, Woodie Zachry, Jean van Wyk, Marisol Martinez

**Affiliations:** 1 Global Health Economics Outcomes Research, AbbVie, North Chicago, IL, USA; 2 Infectious Diseases, St James Hospital, Dublin, Ireland; 3 Infectious Disease Department, Medical Center for Infectious Diseases Berlin (MIB), Berlin, Germany; 4 Clinic of Infectious and Tropical Diseases, San Paolo Hospital, University of Milan, Milan, Italy; 5 Infectious Disease Department, Saint-Petersburg HIV Centre, Saint Petersburg, Russian Federation; 6 Infectious Disease Department, Guangzhou 8th People's Hospital, Guangzhou, China; 7 Infectious Diseases, Helios Salud, Buenos Aires, Argentina; 8 DST/AIDS, Centro de Referência e Treinamento, São Paulo, Brazil; 9 Medical Affairs, AbbVie, North Chicago, IL, USA

## Abstract

**Introduction:**

Global HIV-1 prevalence is 35.3 million [1]; women comprise >50% of those infected. The majority of women may lack regular care and only one-fourth are virologically suppressed [2]. ELLA is a cross-sectional, non-interventional study conducted across Europe, Latin America, Canada and Asia that describes barriers to care for HIV-infected women and associations with disease stage, symptoms and health-related quality of life (HRQoL).

**Methods:**

HIV-infected women eligible for ELLA (≥18 years) completed: Barrier to Care Scale (BACS) comprising 12 items in four domains (Index range 0–12, Overall range 1–4, greater=more barriers, Overall score ≥2 considered severe); AIDS Clinical Trials Group (ACTG) Health Status Assessment comprising 21 items assessing 9 HRQoL domains (range 0–100, greater=better); and ACTG Symptom Distress Module comprising 20 symptoms rated on bother (range 0–4, greater=more bother). Healthcare providers documented medical history and HIV clinical data. Correlations of BACS response and last reported VL/CD4 count with HIV symptoms and HRQoL were analyzed. Spearman rank order was used to test correlations with statistical significance set at p<0.05.

**Results:**

Enrollment: 1931 women from 30 countries; mean age 40 years (16.9% >50 years); 47.7% education <12 years; 36% unemployed; 82.9% urban residence. HIV was acquired heterosexually in 83.0%; 88.2% of subjects were on ART; 57.5% had VL<50 c/ml; mean CD4 was 540.5 c/µL. Mean [SD] BACS Index and Overall scores were 6.19 [3.47] (N=1818) and 2.09 [0.71] (N=1922), respectively. Stigma was a prominent barrier. Lower (better) BACS Index and Overall scores correlated with better HRQoL on all nine domains (p<0.0001). Lower VL and greater CD4 count were both correlated with better HRQoL for eight of nine domains (p<0.04, p≤0.0002, respectively) excepting pain. Lower BACS Index and Overall scores correlated with fewer symptom count and less symptom bother (p<0.0001). Fewer symptom count and less symptom bother correlated with better HRQoL on all nine domains (p<0.0001). While greater CD4 count correlated with fewer HIV symptoms and less bother (p<0.0001), VL did not significantly correlate with either.

**Conclusions:**

In HIV-infected women, reduced barriers to care correlated with fewer symptoms, less symptom bother and better HRQoL. Improved HRQoL may be mediated by greater CD4 counts and fewer symptoms. Better access to care may improve HRQoL outcomes in this population.
